# Risk Factors for Stiff Left Atrial Physiology 1 Year After Catheter Ablation of Atrial Fibrillation

**DOI:** 10.3389/fphys.2021.740600

**Published:** 2021-09-20

**Authors:** Jae-Hyuk Lee, Oh-Seok Kwon, Hee Tae Yu, Tae-Hoon Kim, Jae-Sun Uhm, Boyoung Joung, Moon-Hyoung Lee, Hui-Nam Pak

**Affiliations:** Department of Cardiology, Yonsei University Health System, Seoul, South Korea

**Keywords:** atrial fibrillation, catheter ablation, stiff left atrium, extensive ablation, rhythm outcome

## Abstract

Catheter ablation is the most effective rhythm control method for patients with atrial fibrillation (AF); however, it inevitably causes atrial tissue damage. We previously reported that AF catheter ablation (AFCA) increases left atrial (LA) pressure without changes in symptom scores. We hypothesized that extensive LA ablation increased the risk of stiff LA physiology. We included 1,720 patients (69.1% male, 60.0 [53.0–68.0] years old, 66.2% with paroxysmal AF) who underwent *de novo* AFCA and echocardiography before and 1-year after the procedure. Stiff LA physiology was defined, when the amount of the estimated pulmonary arterial pressure increase between the pre-procedural and the 1-year post-procedural follow-up echocardiography was >10 mmHg and when right ventricular systolic pressure (RVSP) was >35 mmHg at 1-year follow-up echocardiography. The failed rhythm control within 1 year was defined as recurrent AF despite using anti-arrhythmic drugs or cardioversion within a year of AFCA. We explored the incidence and risk factors for stiff LA physiology and the rhythm outcome of AFCA. Among the 1,720 patients, 64 (3.7%) had stiff LA physiology 1 year after AFCA. Stiff LA physiology was independently associated with diabetes (odds ratio [OR], 2.36 [95% CI, 1.14–4.87], *p* = 0.020), the ratio of the peak mitral flow velocity of the early rapid filling to the early diastolic velocity of the mitral annulus (E/Em; OR, 1.04 [95% CI, 1.00–1.10], *p* = 0.049), LA pulse pressure (Model 2: OR, 1.05 [95% CI, 1.00–1.11], *p* = 0.049), low LA voltage (OR, 0.36 [95% CI, 0.18–0.74], *p* = 0.005), empirical extra-pulmonary vein (PV) LA ablation (OR, 2.60 [95% CI, 1.17–5.74], *p* = 0.018), and radiofrequency (RF) ablation duration (Model 2: OR, 1.02 [95% CI, 1.01–1.03], *p* = 0.003). Although the incidence of post-AFCA stiff LA physiology was 3.7% and most of the cases were subclinical, the empirical extra-PV ablation was associated with this undesirable condition. In addition, patients who had low mean LA voltage before AFCA could be susceptible to stiff LA physiology.

## Introduction

Catheter ablation is the most effective rhythm control method for patients with atrial fibrillation (AF), and various clinical benefits of catheter ablation have been reported, including a reduction in the mortality rate in patients with heart failure (Marrouche et al., 2018). However, it is a destructive procedure involving heating or freezing as the energy source for AF catheter ablation (AFCA); this inevitably causes atrial tissue damage, resulting in desiccation necrosis, fibrosis, and scar generation (Nath et al., 1994; Cesario et al., 2007). In particular, atrial substrate modification, such as an empirical or targeted extra-pulmonary vein (PV) ablation, has been performed in patients with advanced AF and significant atrial structural remodeling. Park et al. reported an elevated left atrial (LA) pressure and stiffness at the time of repeat ablation as compared to *de novo* procedures (Park et al., 2019). They found that the level of the increase in LA pressure was more significant in patients who underwent an empirical extra-PV LA ablation than in those who underwent a circumferential PV isolation (CPVI) alone. However, the symptom score did not differ in that study (Park et al., 2019). In addition, patients with AF with higher LA pressure, stiffness, or wall stress had a higher recurrence rate after AFCA (Park et al., 2014, 2015; Lee et al., 2021a). Stiff LA syndrome is a form of symptomatic pulmonary arterial (PA) hypertension, which is caused by a decreased LA function after mitral valve surgery (Pilote et al., 1988). Recently, it has been reported that stiff LA syndrome can occur after extensive AFCA. However, little is known about the frequency and mechanism of the post-AFCA stiff LA physiology (Gibson et al., 2011; Witt et al., 2014). As previous studies that compared the LA pressure before and after AFCA measured the invasive LA pressure only in patients who underwent repeat ablation, there might be a selection bias (Park et al., 2019). Therefore, in this study, we explored the incidence and clinical features of stiff LA physiology in all patients who underwent *de novo* AFCA using 1-year follow-up echocardiographic parameters. The purpose of this study was to identify the incidence and clinical predictors associated with the development of stiff LA physiology after AFCA and to evaluate whether it was particularly related to the empirical extra-PV LA ablation. We applied the previously reported echocardiographic definition of stiff LA physiology (Witt et al., 2014).

## Materials and Methods

### Study Population

The study protocol adhered to the principles of the Declaration of Helsinki and was approved by the Institutional Review Board of the Yonsei University Health System. All patients provided written informed consent for inclusion in the Yonsei AF Ablation cohort. A total of 1,720 consecutive patients who underwent *de novo* AFCA between March 2009 and January 2020 in a single center were prospectively enrolled in this study. The comorbidities were gathered from the medical records at the time of AFCA. Heart failure was defined according to the guidelines (Writing Committee et al., 2016). In all patients, LA pressure was measured during the procedure, and echocardiography with measurement of the right ventricular systolic pressure (RVSP) was conducted before and 1 year after AFCA. The patients were divided into two groups based on the occurrence of stiff LA physiology using pre- and post-AFCA echocardiographic estimation of PA pressure. The exclusion criteria were as follows: (1) AF refractory to electrical cardioversion; (2) no available data on RVSP on echocardiography before or after AFCA; (3) RVSP >40 mmHg on the echocardiography conducted before the AFCA; (4) repeat ablation within a year after *de novo* procedure; (5) AF with rheumatic valvular disease; (6) patients who had PV stenosis; and (7) prior AF ablation or cardiac surgery. All patients stopped all anti-arrhythmic drugs (AADs) for a period corresponding to at least five half-lives before AFCA.

### Echocardiography Follow-Up and Definition of Stiff LA Physiology

Transthoracic echocardiography (TTE) was conducted within 3 months prior to the procedure and at the 1-year follow-up. PA systolic pressure was estimated using RVSP on echocardiography. RVSP was calculated from the peak tricuspid regurgitant jet velocity (V) using the modified Bernoulli's equation (RVSP = 4V^2^ + right atrial pressure). Stiff LA physiology was defined, when the amount of the estimated increase in PA pressure between the pre-procedural and the 1-year post-procedural follow-up echocardiography was >10 mmHg and when RVSP was >35 mmHg at 1-year follow-up echocardiography (Witt et al., 2014). The interobserver and intraobserver reliability for the RVSP on echocardiography were 92 and 95%, respectively.

### Measurement of LA Pressure, LA Wall Thickness, and LA Wall Stress

During the AFCA procedure, LA pressure was measured during sinus rhythm and AF immediately after a transseptal puncture, as described in the previous studies (Park et al., 2014, 2019). If the initial rhythm was AF, we measured LA pressure during sinus rhythm after terminating AF by internal cardioversion, followed by a waiting period of at least 3 min to allow for recovery from atrial stunning from cardioversion (Park et al., 2014, 2019). We excluded patients in whom LA pressure during sinus rhythm could not be measured due to frequent reinitiation of AF after electrical cardioversion.

We developed a customized software (AMBER, Laonmed Inc., Seoul, Korea) that measured the LA wall thickness by applying Laplace's equation in the cardiac CT images (Kwon et al., 2020; Lee et al., 2021b). The CT scan was conducted within a month before the AFCA. The spatial resolutions of the CT images were within 0.3–0.55 mm for the x- and y-axes, and the slice thickness of the z-axis was 0.5 mm. The spatial resolution of the CT was set to the normalized vector in the 3D Euclidean space. The methods and principles of the customized software (AMBER) were previously described in detail, and the results have been well validated with a 3D printed phantom model in 120 patients (Kwon et al., 2020; Lee et al., 2021b). In brief, the endocardium of the LA was semiautomatically divided on the cardiac CT using an edge detector. Then, the LA wall was extracted with an overlapped area by the morphological operations after separation from other tissues using the multi-Otsu threshold algorithm in a histogram of Hounsfield units. The LA wall thickness was measured by applying Laplace's equation and Euler's method in 3D space.

The LA wall stress (LAW-stress) (dyn/cm^2^) was calculated using the Law of Laplace [σ = (P × *r*)/2*h* (σ, wall stress; P, pressure; r, radius; h, wall thickness)] (Falsetti et al., 1970; Wang et al., 2011). The peak LA pressure during sinus rhythm was directly measured during the AF procedure, and the LA radius was defined as half of the LA anterior-posterior (AP) diameter with TTE. Therefore, LAW-stress was calculated using the following equation: LAW-stress = (peak LA pressure × LA AP diameter)/(4 × LA wall thickness). LAW-stress was expressed as dyn/cm^2^ (1 mmHg = 1,333 dyn/cm^2^). We previously reported that the LAW-stress calculated using the abovementioned equation is a useful prognostic parameter for AF recurrence after AFCA (Lee et al., 2021a).

### Electrophysiological Studies and Catheter Ablation

**Figure 1:**
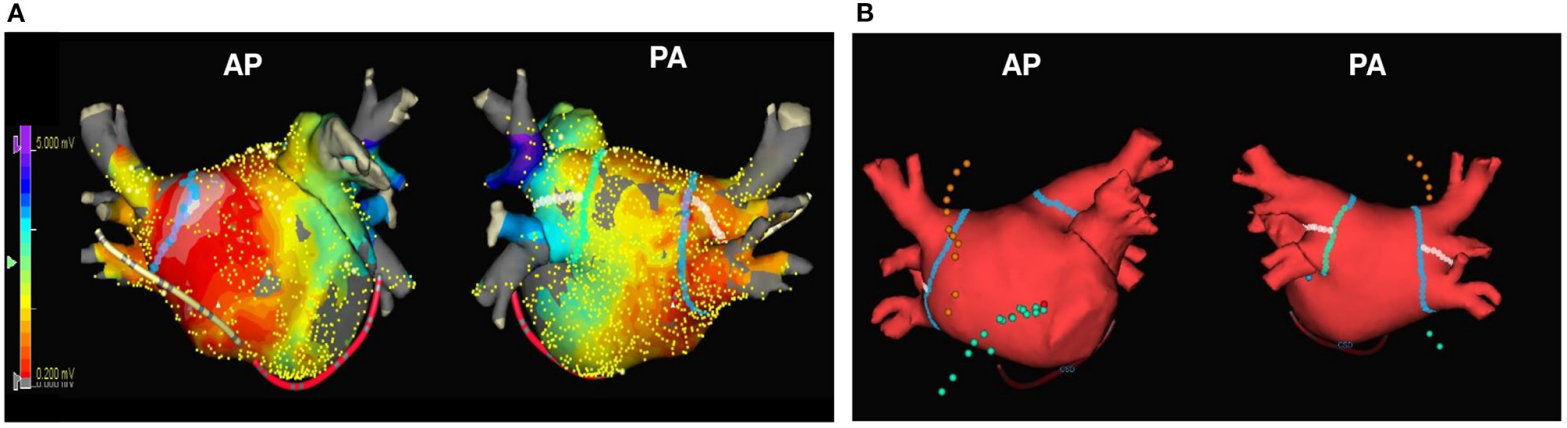
The representative images of the LA voltage map and usual ablation lesion set. The representative images of LA voltage map **(A)** and usual ablation lesion set that include CPVI, SVC-right septal line, and CTI line **(B)**. CPVI, circumferential pulmonary vein isolation; CTI, cavo-tricuspid isthmus; LA, left atrium; SVC, superior vena cava.

The electrophysiological mapping method and the AFCA technique/strategy used during the study period were consistently performed as described in a previous study (Yu et al., 2017). In brief, an open irrigated-tip catheter (Celsius, ThermoCool SF [Johnson & Johnson Inc., Diamond Bar, CA, USA] or CoolFlex [St. Jude Medical Inc., Minnetonka, MN, USA]; 30–35 W, 45°C) was used to deliver radiofrequency (RF) energy for ablation under 3D electroanatomical mapping (NavX [St. Jude Medical, Minnetonka, MN, USA] or CARTO3 [Johnson & Johnson Inc.]) merged with 3D spiral CT. For high-quality voltage maps, LA electrogram voltage maps were generated using a circumferential mapping catheter during high right atrial pacing at 500 ms before CPVI. However, in a minority of the patients with recurrent AF at the beginning of the procedure, we acquired voltage maps during sinus rhythm after the completion of CPVI. To avoid any false detection of inadequate voltages, the Automap module, which is the system setting for a high-quality voltage map, was used during the map acquisition. In brief, we set the criteria of components, such as the morphology of original template beat, cycle length, catheter moving speed, and signal-to-noise threshold, for adequate point in the Automap module, and the system could discriminate adequate contact electrogram from inadequate mapping point, such as noise. We obtained the peak-to-peak amplitude of contact bipolar electrograms from 500 to 1,000 points on the LA endocardium, and the mean LA electrogram voltage was calculated. If frequently recurring AF persisted after three attempts at cardioversion, no further efforts were made to generate an LA voltage map. All patients initially underwent a CPVI. For patients with persistent AF, roof line, posterior-inferior line, anterior line, cavotricuspid isthmus line, superior vena cava to the septal line, or complex fractionated atrial electrogram-guided ablation, were added at the discretion of the operator. The procedure was considered complete when there was no immediate recurrence of AF after cardioversion with isoproterenol infusion (5–10 μg/min; target heart rate, 120 bpm). In the case of mappable AF triggers, extra-PV foci were mapped and ablated as much as possible. Although we have kept consistent ablation protocol used by experienced operators, the catheter technology and mapping technologies kept changing during the long period of enrollment (Park JW, CircJ2019). We used contact force catheters in 11.6% of the patients enrolled, and extra-PV ablation was dependent on the study protocol or discretion of the operators. Systemic anticoagulation was achieved with intravenous heparin while maintaining an activated clotting time of 350–400 s during the procedure. Representative images of the bipolar voltage map and usual ablation lesion set are presented in Figure 1.

### Post-ablation Management and Follow-Up

All patients visited the scheduled outpatient clinic at 1, 3, 6, and 12 months after AFCA and every 6 months thereafter or whenever symptoms occurred. All patients underwent electrocardiography at every visit, as well as 24 h Holter recording at 3 and 6 months, then every 6 months for 2 years, annually for 2–5 years, and then biannually after 5 years, following the modified 2012 HRS/EHRA/ECAS expert consensus statement guidelines (Calkins et al., 2012). Whenever patients reported palpitations, Holter monitor or event monitor recordings were obtained and evaluated to check for the recurrence of arrhythmias. AF recurrence was defined as any episodes of AF or atrial tachycardia lasting for at least 30 s. Any electrocardiographic documentation of AF recurrence 3 months after the blanking period was identified as clinical recurrence. We defined a “failed rhythm control” as recurrent AF despite AAD or cardioversion within a year of AFCA.

### Statistical Analysis

Continuous variables were expressed as the mean ± SD for normally distributed variables and as the median with the interquartile range for non-normally distributed variables; they were compared using the Student's *t*-test and the Wilcoxon rank-sum test, respectively. The categorical variables were reported as counts (percentages) and were compared using the chi-square or Fisher's exact test. The echocardiographic parameters before and 1 year after the procedure were compared using a paired *t*-test. The logistic regression analysis was used to identify risk factors for stiff LA physiology after AFCA and to estimate the odds ratios (ORs), 95% CIs, and *p*-values. The variables selected for the multivariate analysis were those with a *p*-value < 0.05 on the univariate analysis. Since there were variables that had multicollinearity, two logistic regression models were investigated separately. The Kaplan-Meier analysis with log-rank test was used to analyze the probability of freedom from AF recurrences after AFCA. The Statistical Package for the Social Sciences (SPSS) version 25.0 for Windows (IBM Corporation, Armonk, NY, USA) and the R software version 3.6.2 (The R Foundation for Statistical Computing, Vienna, Austria) were used for the data analysis.

## Results

### Incidence of Stiff LA Physiology and Clinical Characteristics

**Figure 2:**
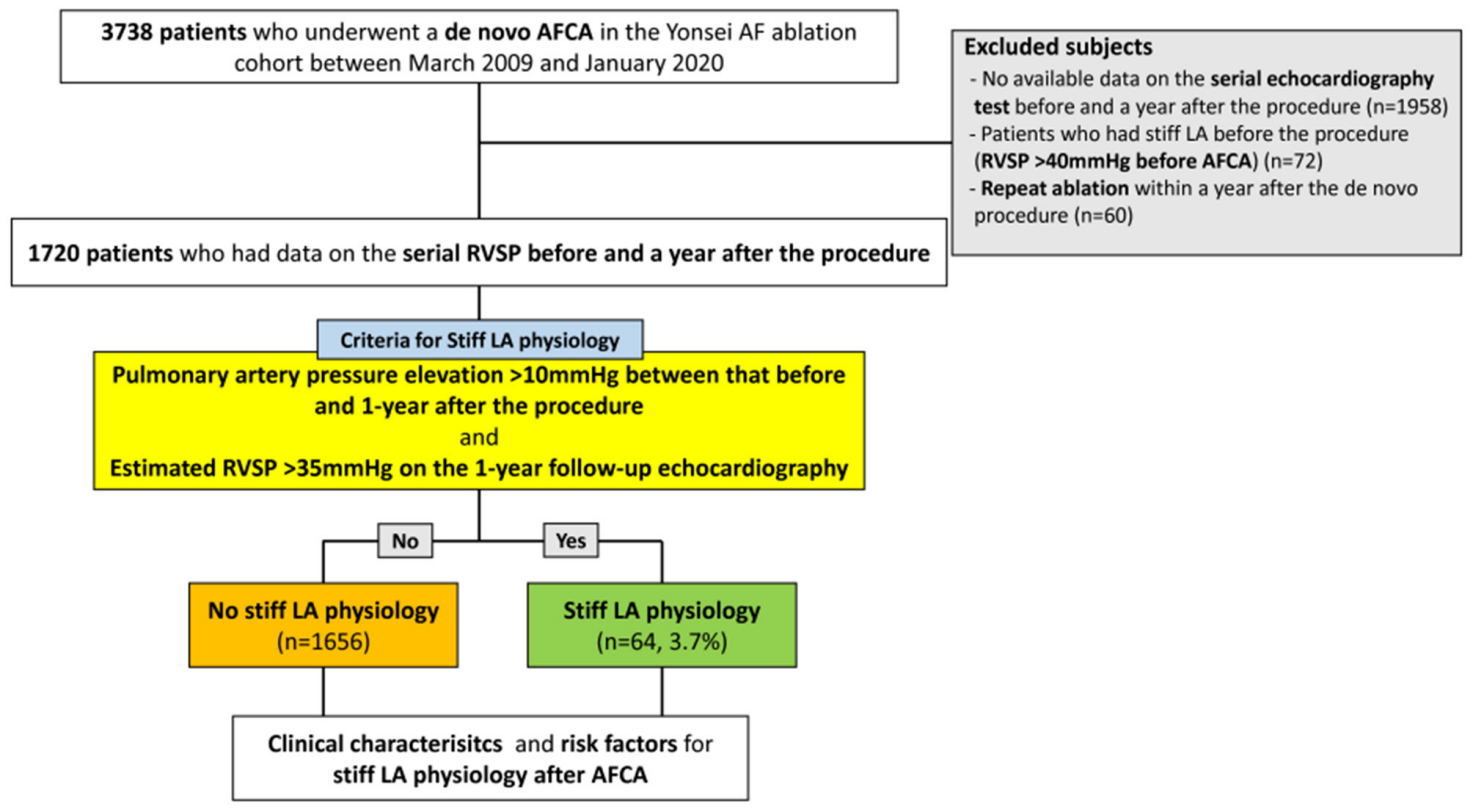
Flow chart of the study. AFCA, atrial fibrillation catheter ablation; LA, left atrial or left atrium; RVSP, right ventricular systolic pressure.

A total of 1,720 patients (69.1% male, 60.0 [53.0–68.0] years old, 66.2% with paroxysmal AF) who underwent *de novo* AFCA and echocardiography before and 1 year after the procedure were enrolled in this study. We found stiff LA physiology in 64 (3.7%) out of the 1,720 patients 1 year after the *de novo* procedure (Figure 2). The patients who had stiff LA physiology after ablation were older (*p* < 0.001) and had diabetes (*p* = 0.008), higher proportion of persistent AF (*p* = 0.001), and higher CHA_2_DS_2_-VASc score (*p* = 0.001) than their counterparts. LA peak pressure (*p* = 0.005) and LAW-stress (*p* = 0.001) were higher, and mean LA voltage was lower (*p* = 0.001) in patients with stiff LA physiology (Table 1).

**Table 1:** Baseline characteristics according to the occurrence of stiff LA physiology.

	**All subjects (n = 1,720)**	**No stiff LA physiology (*n* = 1,656)**	**Stiff LA physiology (*n* = 64)**	**P**
Persistent AF, *n* (%)	582 (33.8)	548 (33.1)	34 (53.1)	0.001
AF duration (month)	24.0 (9.0–49.0)	24.0 (9.0–49.5)	23.0 (7.0–48.0)	0.893
Age (years)	60.0 (53.0–68.0)	60.0 (53.0–67.0)	64.5 (59.0–74.0)	<0.001
Male, *n* (%)	1188 (69.1)	1148 (69.3)	40 (62.5)	0.307
Comorbidity, *n* (%)				
Hypertension	834 (48.5)	798 (48.2)	36 (56.2)	0.255
Diabetes mellitus	268 (15.6)	250 (15.1)	18 (28.1)	0.008
Stroke/TIA	217 (12.6)	208 (12.6)	9 (14.1)	0.87
Vascular disease	229 (13.3)	215 (13.0)	14 (21.9)	0.062
Heart failure	234 (13.6)	220 (13.3)	14 (21.9)	0.075
Body mass index (kg/m^2^)	24.6 (22.9–26.5)	24.6 (22.9–26.5)	24.8 (23.1–27.1)	0.595
Body surface area (m^2^)	1.8 (1.7–1.9)	1.8 (1.7–1.9)	1.8 (1.6–1.9)	0.120
CHA_2_DS_2_-VASc score	2.0 (1.0–3.0)	2.0 (1.0–3.0)	2.0 (1.0–3.5)	0.001
LAP, peak (mmHg)	20.0 (15.0–27.0)	20.0 (15.0–27.0)	23.0 (19.0–33.0)	0.005
LAP, nadir (mmHg)	4.0 (1.0–8.0)	4.0 (1.0–8.0)	6.0 (0–10.5)	0.302
LA pulse pressure (mmHg)	16.0 (11.0–21.0)	16.0 (11.0–21.0)	17.0 (12.5–24.5)	0.049
Mean LA voltage (mV)	1.3 (0.8–1.8)	1.3 (0.8–1.8)	0.8 (0.6–1.4)	<0.001
Mean LA wall thickness (mm)	1.9 (1.8–2.1)	1.9 (1.8–2.1)	2.0 (1.8–2.1)	0.679
Mean LAW-stress (10^3^ dyn/cm^2^)	145.1 (100.5–203.9)	143.7 (99.6–201.2)	191.8 (120.4–266.2)	0.001
Post-procedural medication, *n* (%)				
ACEi or ARB	607 (35.3)	578 (34.9)	29 (45.3)	0.117
Beta blocker	610 (35.5)	589 (35.6)	21 (32.8)	0.745
Anti-arrhythmic agent	255 (14.9)	244 (14.9)	11 (17.2)	0.737

*Values are presented as median (Q1–Q3 quartiles [25th and 75th percentiles]) or number (%)*.

*ACEi, angiotensin-converting enzyme inhibitor; AF, atrial fibrillation; ARB, angiotensin II receptor blocker; LA, left atrial; LAP, left atrial pressure; LAW-stress, left atrial wall stress; PV, pulmonary vein; TIA, transient ischemic attack*.

### Echocardiographic and Procedural Characteristics in Patients With Stiff LA Physiology

**Figure 3:**
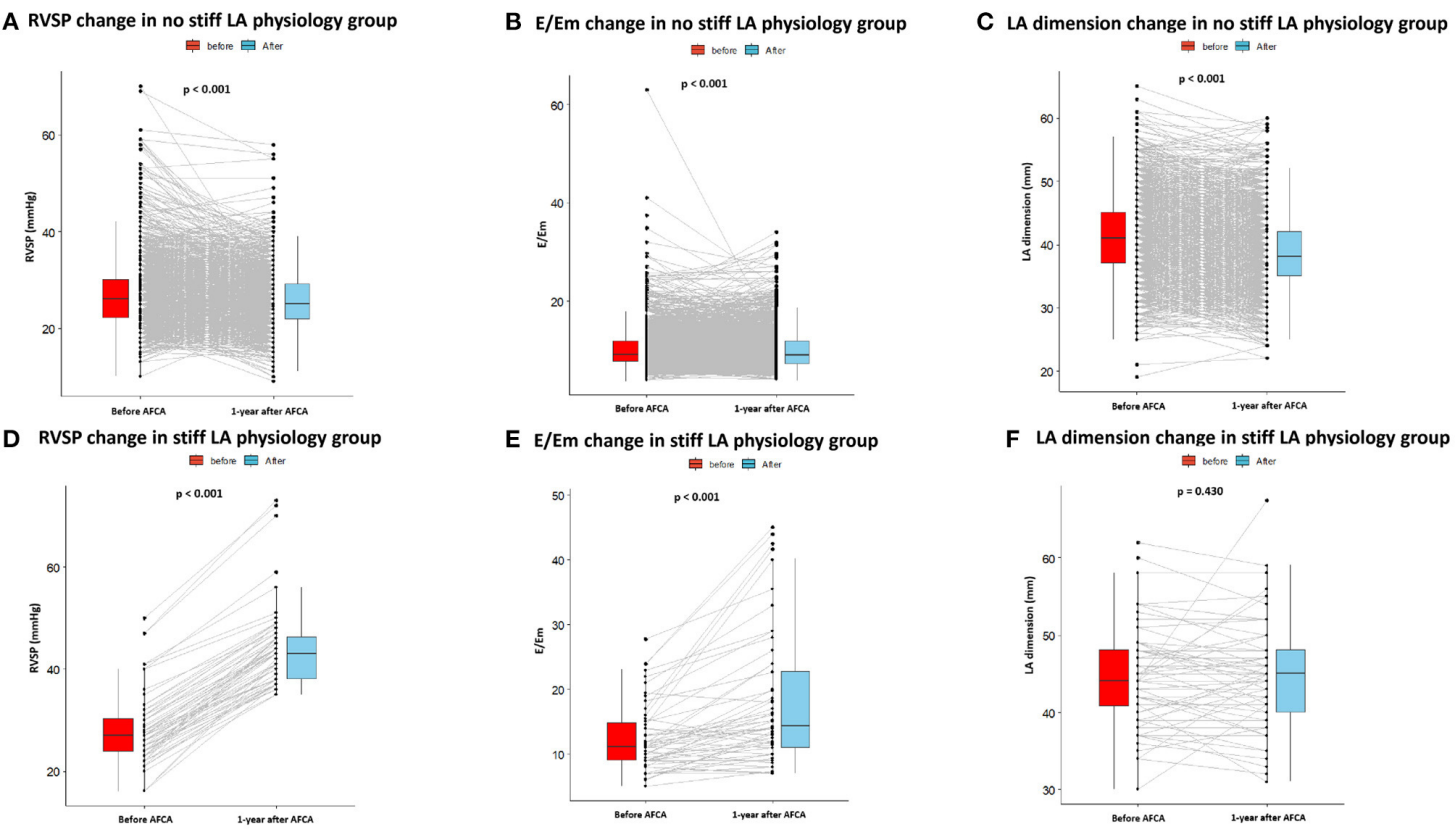
Changes in echocardiography parameters before and 1 year after AFCA depending on the development of stiff LA physiology. In patients without stiff LA physiology, RVSP **(A)**, E/Em **(B)**, and LA dimension **(C)** significantly decreased 1 year after AFCA. In contrast, in patients with stiff LA physiology, RVSP **(D)** and E/Em **(E)** increased, and LA dimension **(F)** did not change. E/Em, the ratio of the peak mitral flow velocity of the early rapid filling to the early diastolic velocity of the mitral annulus; LA, left atrial or left atrium; RVSP, right ventricular systolic pressure.

Patients who had stiff LA physiology after AFCA had higher LA dimension (*p* < 0.001), LA volume index (*p* < 0.001), and the ratio of the peak mitral flow velocity of the early rapid filling to the early diastolic velocity of the mitral annulus (E/Em) (*p* < 0.001) than those without stiff LA physiology at both the pre-procedural and follow-up TTE (Table 2). Figure 3 presents changes in echocardiographic parameters 1 year after the procedures, depending on the development of stiff LA physiology. While RVSP (*p* < 0.001, Figure 3A), E/Em (*p* < 0.001, Figure 3B), and LA dimension (*p* < 0.001, Figure 3C) significantly decreased in the majority of patients without stiff LA physiology, RVSP (*p* < 0.001, Figure 3D) and E/Em (*p* < 0.001, Figure 3E) increased, and LA dimension (*p* = 0.430, Figure 3F) did not change in the stiff LA physiology group. The stiff LA physiology group had a longer procedure (*p* < 0.001) and RF ablation (*p* < 0.001) times and underwent the empirical extra-PV LA ablations more often (34.8% vs. 57.8%, *p* < 0.001, Table 2).

**Table 2:** Echocardiographic and procedural characteristics according to the occurrence of stiff LA physiology.

	**All subjects (*n* = 1,720)**	**No stiff LA physiology (*n* = 1,656)**	**Stiff LA physiology (*n* = 64)**	**P**
Pre-procedural TTE				
LA dimension (mm)	41.0 (37.0–46.0)	41.0 (37.0–45.0)	44.0 (40.5–48.0)	<0.001
LA volume index (ml/m^2^)	35.9 (28.9–44.6)	35.7 (28.7–44.5)	40.6 (34.9–53.5)	<0.001
LV ejection fraction (%)	64.0 (59.0–68.0)	64.0 (59.0–68.5)	63.0 (57.0–67.5)	0.253
E/Em	9.4 (8.0–12.0)	9.3 (7.9–12.0)	11.1 (9.0–14.8)	0.002
TR jet (m/s)	2.3 (2.1–2.5)	2.3 (2.1–2.5)	2.3 (2.1–2.5)	0.269
RVSP (mmHg)	26.0 (22.0–30.0)	26.0 (22.0–30.0)	27.0 (23.5–30.5)	0.238
1-year f/u TTE				
LA dimension (mm)	39.0 (35.0–43.0)	38.0 (35.0–42.0)	45.0 (40.0–48.0)	<0.001
LA volume index (ml/m^2^)	29.4 (24.1–37.8)	29.1 (23.9–36.9)	40.6 (35.3–57.3)	<0.001
LV ejection fraction (%)	65.0 (61.0–69.0)	65.0 (61.0–69.0)	65.0 (60.5–71.0)	0.829
E/Em	9.3 (7.5–12.5)	9.1 (7.4–12.0)	14.3 (11.0–22.8)	<0.001
TR jet (m/s)	2.2 (2.0–2.5)	2.2 (2.0–2.4)	3.0 (2.8–3.2)	<0.001
RVSP (mmHg)	25.0 (22.0–29.0)	25.0 (21.5–29.0)	43.0 (38.0–46.5)	<0.001
RF ablation duration (min)	77.2 (58.8–96.9)	76.8 (58.5–95.9)	89.9 (74.3–118.0)	<0.001
Procedure time (min)	176.0 (145.0–210.0)	176.0 (144.0–209.0)	197.0 (163.0–233.5)	<0.001
Ablation lesion, *n* (%)				
CPVI	1,720 (100)	1,656 (100)	64 (100)	1
Roof line	548 (31.9)	513 (31.0)	35 (54.7)	<0.001
Posterior-inferior line	480 (27.9)	449 (27.1)	31 (48.4)	<0.001
POBI	471 (27.4)	440 (26.6)	31 (48.4)	<0.001
Anterior line	449 (26.1)	413 (24.9)	36 (56.2)	<0.001
Left lateral isthmus	75 (4.4)	70 (4.2)	5 (7.8)	0.289
CFAE ablation	86 (5.0)	77 (4.7)	9 (14.1)	0.002
Empirical extra-PV LA ablation	613 (35.7)	576 (34.8)	37 (57.8)	<0.001
Extra PV foci, *n* (%)	137 (10.6)	134 (10.7)	3 (6.5)	0.503
Failed rhythm control within a year^*^	169 (9.8)	153 (9.2)	16 (25.0)	<0.001
Early recurrence, *n* (%)	508 (29.5)	483 (29.2)	25 (39.1)	0.118
Clinical recurrence, *n* (%)	675 (39.2)	639 (38.6)	36 (56.2)	0.007

*Values are presented as median (Q1–Q3 quartiles [25th and 75th percentiles]) or number (%)*.

* *Failed rhythm control within a year was defined when AF rhythm was recurred and maintained within a year after the procedure even though repeat ablation, anti-arrhythmic drug (AAD), or electrical cardioversion were performed*.

*CFAE, complex fractionated atrial electrograms; CPVI, circumferential pulmonary vein isolation; E/Em, the ratio of the peak mitral flow velocity of the early rapid filling to the early diastolic velocity of the mitral annulus; LA, left atrial; LV, left ventricular; POBI, posterior box isolation; PV pulmonary vein; RF, radiofrequency; RVSP, right ventricular systolic pressure; TR, tricuspid regurgitant*.

### Risk Factors of Stiff LA Physiology After AFCA and Rhythm Outcome

We investigated the factors associated with stiff LA physiology using the multivariate logistic regression analysis (Table 3). We used two different models because RF ablation duration and empirical extra-PV LA ablation had multicollinearity with each other as variables for adjustment. Stiff LA physiology was independently associated with diabetes (OR, 2.36 [95% CI, 1.14–4.87], *p* = 0.020), E/Em (OR, 1.04 [95% CI, 1.00–1.10], *p* = 0.049), LA pulse pressure (Model 2: OR, 1.05 [95% CI, 1.00–1.11], *p* = 0.049), low LA voltage (OR, 0.36 [95% CI 0.18–0.74], *p* = 0.005), empirical extra-PV LA ablation (OR, 2.60 [95% CI, 1.17–5.74], *p* = 0.018), and RF ablation duration (Model 2: OR, 1.02 [95% CI, 1.01–1.03], *p* = 0.003). Furthermore, the low mean LA voltage (OR, 0.47 [95% CI, 0.32–0.67], *p* < 0.001) and the presence of stiff LA physiology (OR, 3.19 [95% CI, 1.64–6.22], *p* = 0.001) were independently associated with failed rhythm control within 1 year (Table 4).

**Table 3:** Logistic regression analysis for stiff LA physiology.

	**Univariate**		**Multivariate model 1^†^**		**Multivariate model 2^†^**	
	**OR (95% CI)**	**P**	**OR (95% CI)**	**P**	**OR (95% CI)**	**P**
Persistent AF	2.29 (1.39–3.78)	0.001	0.77 (0.34–1.71)	0.516	0.81 (0.37–1.77)	0.604
AF duration	1.00 (0.99–1.01)	0.812				
Male	0.74 (0.44–1.24)	0.248				
Age	1.06 (1.03–1.08)	<0.001	1.01 (0.98–1.05)	0.464	1.02 (0.98–1.05)	0.370
Body surface area	0.31 (0.08–1.25)	0.100				
Body mass index	1.04 (0.96–1.13)	0.382				
Comorbidity						
Hypertension	1.38 (0.84–2.29)	0.207				
Diabetes mellitus	2.20 (1.26–3.86)	0.006	2.36 (1.14–4.87)	0.020	2.52 (1.21–5.24)	0.013
Stroke/TIA	1.14 (0.55–2.34)	0.723				
Heart failure	1.83 (0.99–3.36)	0.052				
Vascular disease	1.88 (1.02–3.45)	0.043	1.93 (0.88–4.21)	0.100	1.52 (0.69–3.36)	0.298
Echocardiography						
LA dimension	1.08 (1.04–1.12)	<0.001	1.02 (0.96–1.09)	0.438	1.02 (0.95–1.08)	0.635
LV ejection fraction	0.99 (0.96–1.01)	0.254				
E/Em	1.07 (1.03–1.11)	0.001	1.04 (1.00–1.10)	0.049	1.04 (1.00–1.09)	0.050
TR jet	0.99 (0.89–1.1)	0.880				
RVSP	1.02 (0.98–1.05)	0.334				
LAP, peak^*^	1.04 (1.02–1.07)	<0.001				
LAP, nadir^*^	1.04 (0.99–1.08)	0.091				
LA pulse pressure^*^	1.04 (1.02–1.07)	0.002	1.05 (1.00–1.10)	0.060	1.05 (1.00–1.11)	0.049
Procedure time (min)	1.01 (1.00–1.01)	<0.001				
RF ablation duration (min)^†^	1.02 (1.01–1.03)	<0.001			1.02 (1.01–1.03)	0.003
Empirical extra-PV LA ablation^†^	2.56 (1.54–4.25)	<0.001	2.60 (1.17–5.74)	0.018		
Extra PV foci	0.58 (0.18–1.89)	0.366				
Post-procedural medication						
ACEi/ARB	1.54 (0.93–2.55)	0.091				
Beta-blocker	0.88 (0.52–1.50)	0.646				
Anti-arrhythmic drug	1.19 (0.61–2.31)	0.607				
Mean LA voltage	0.35 (0.20–0.59)	<0.001	0.36 (0.18–0.74)	0.005	0.33 (0.16–0.68)	0.003
Mean LA wall thickness	0.81 (0.39–1.71)	0.585				
Mean LAW-stress	1.00 (1.00–1.01)	<0.001	1.00 (0.99–1.00)	0.076	0.99 (0.99–1.00)	0.054

* *LA pulse pressure was included in the multivariate analysis due to multicollinearity among three variables*.

† *Two multivariate models were separately presented because RF ablation duration and extra-PV LA lesion had multicollinearity to each other*.

*ACEi, angiotensin-converting enzyme inhibitor; AF, atrial fibrillation; ARB, angiotensin II receptor blocker; E/Em, the ratio of the peak mitral flow velocity of the early rapid filling to the early diastolic velocity of the mitral annulus; LA, left atrial; LAP, left atrial pressure; LAW-stress, left atrial wall stress; LV, left ventricular; OR, odds ratio; PV, pulmonary vein; RF, radiofrequency; RVSP, right ventricular systolic pressure; TIA, transient ischemic attack; TR, tricuspid regurgitant*.

**Table 4:** Logistic regression analysis for failed rhythm control within 1 year.

	**Univariate**		**Multivariate**	
	**OR (95% CI)**	**P**	**OR (95% CI)**	**P**
Persistent AF	1.88 (1.36–2.59)	<0.001	1.10 (0.71–1.69)	0.674
Male	1.40 (0.97–2.01)	0.073		
Age	1.00 (0.98–1.01)	0.874		
Hypertension	1.41 (1.02–1.94)	0.035	1.27 (0.85–1.89)	0.237
Diabetes mellitus	0.98 (0.63–1.53)	0.941		
Heart failure	1.00 (0.63–1.59)	0.998		
LA dimension	1.04 (1.02–1.07)	0.001	1.01 (0.98–1.05)	0.514
LV ejection fraction	1.01 (0.99–1.02)	0.584		
E/Em	0.99 (0.95–1.03)	0.578		
Mean LA voltage	0.41 (0.29–0.58)	<0.001	0.47 (0.32–0.67)	<0.001
Mean LA wall thickness	0.72 (0.45–1.15)	0.173		
Mean LA wall stress	1.00 (1.00–1.00)	0.422		
Stiff LA physiology	3.27 (1.82–5.91)	<0.001	3.19 (1.64–6.22)	0.001

*AF, atrial fibrillation; E/Em, ratio of the peak mitral flow velocity of the early rapid filling to the early diastolic velocity of the mitral annulus; LA, left atrial; LV, left ventricular; OR, odds ratio*.

**Figure 4:**
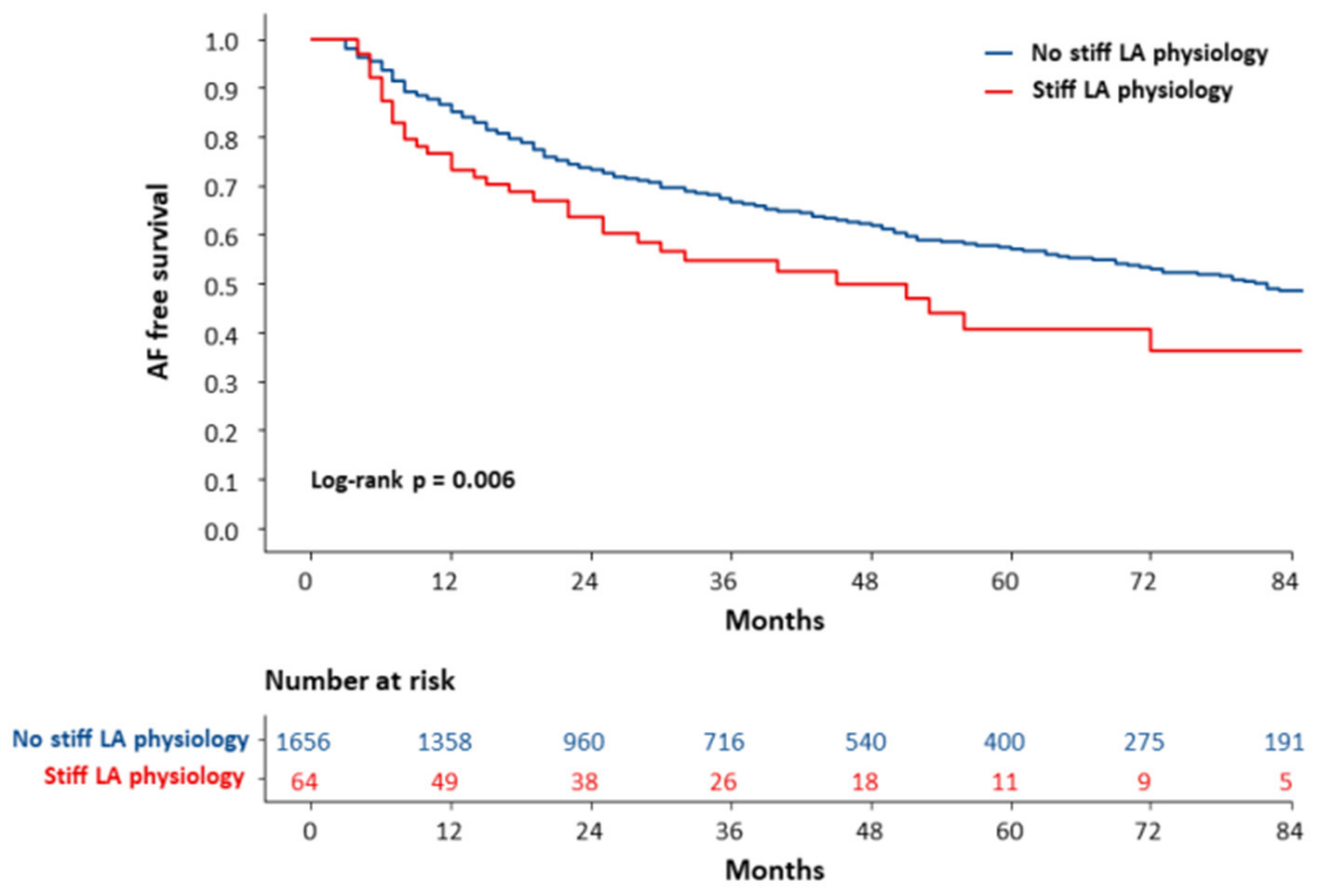
The Kaplan-Meier curve for clinical recurrence of AF according to the development of stiff LA physiology. The clinical recurrence of AF was worse in patients with stiff LA physiology (log-rank *p* = 0.006). AF, atrial fibrillation; LA, left atrial or left atrium; RVSP, right ventricular systolic pressure.

During 28 (14.0–56.0) months of follow-up, the clinical recurrence (*p* = 0.007) rates were significantly higher in patients who had stiff LA physiology 1 year after AFCA (Table 2). On the Kaplan-Meier analysis, rhythm outcome was worse in patients with stiff LA physiology (log-rank *p* = 0.002) (Figure 4).

## Discussion

### Main Findings

In this study, we evaluated the incidence and risk factors of stiff LA physiology 1 year after AFCA using echocardiographic estimated RVSP and PA pressure. Stiff LA physiology occurred in 3.7% of patients after *de novo* AFCA. Stiff LA physiology was independently associated with diabetes, left ventricular (LV) diastolic function, and low LA voltage. Empirical extra-PV ablation was an independent predictor of stiff LA physiology. In addition, rhythm outcomes were worse in patients with stiff LA physiology. Although the incidence of post-AFCA stiff LA physiology was low and most of the cases were subclinical, the empirical extra-PV ablation contributed to this undesirable condition.

### Stiff LA Physiology After Cardiovascular Interventions

Stiff LA syndrome was first described in patients who had developed pulmonary hypertension after undergoing mitral valve surgery, and this concept has recently been applied to patients after AFCA procedures (Pilote et al., 1988; Gibson et al., 2011). Park et al. (2014, 2015) previously reported that increased LA pressure and reduced compliance after AFCA were associated with advanced LA substrate remodeling and worse rhythm outcomes. Although empirical extra-PV ablation increases LA pressure more significantly than CPVI alone, the symptom score did not differ in our recent study (Park et al., 2019). Small LA diameter, diabetes, and obstructive sleep apnea were also reported as risk factors for stiff LA syndrome (Gibson et al., 2011). A previous study reported that a severe LA scar, defined as an area with low voltage, was associated with stiff LA syndrome (Gibson et al., 2011). This study also showed that low mean LA voltage at baseline was independently associated with stiff LA physiology. It might be explained that patients who had more advanced structural remodeling before the procedure would be susceptible to stiff LA physiology after AFCA. Although the mechanism is unclear, diabetes which accompanies inflammation and fibrosis was an independent predictor of stiff LA physiology in this study. The prevalence of failed rhythm control within a year was higher in the stiff LA physiology group (*p* < 0.001, Table 2), and the stiff LA physiology (OR, 3.19 [1.64–6.22], *p* = 0.001) was also the independent factor of failed rhythm control within a year (Table 4). However, it was hard to conclude a causal-result relationship between stiff LA physiology and failed rhythm control within a year because we determined both parameters simultaneously 1 year after the procedure. We found E/Em elevation, which reflected worsening of LV diastolic function, among patients with stiff LA physiology. LV diastolic dysfunction increases PA pressure and RVSP and is attributed to stiff LA physiology (Shoemaker et al., 2011; Witt et al., 2014). A reduced reservoir or pump function in stiff LA physiology inversely has a negative effect on the LV filling pressure, resulting in increased E/Em (Appleton et al., 1988).

### Empirical Extra-PV LA Ablations in AFCA

Although AFCA is an effective AF rhythm control method, various extra-PV substrate modifications have been attempted to reduce the substantial and continuous recurrence rate, especially after persistent AF ablation (Jais et al., 2004; Haissaguerre et al., 2005; Willems et al., 2006; Knecht et al., 2008). However, the recently attempted randomized clinical trials have failed to prove the benefits of empirical extra-PV ablation in terms of the AFCA rhythm outcome (Verma et al., 2015; Lee et al., 2019). In addition, the current guidelines do not recommend routine empirical extra-PV ablation (Calkins et al., 2017). The untargeted empirical substrate ablation may generate new scars that are correlated with LA function (Wylie et al., 2008): the more the touches, the more the scars. We previously reported that empirical extra-PV LA ablation increased LA pressure and stiffness without the aggravation of symptom scores compared with CPVI alone (Park et al., 2019). In this study, the empirical extra-PV LA ablation and the long duration of RF ablation were consistently associated with stiff LA physiology on follow-up echocardiography. Therefore, appropriate mapping and ablating extra-PV foci, which is the main cause of the long-term AF recurrence after AFCA, are still challenging issues.

### Study Limitations

This study had several limitations. First, this was an observational prospective cohort study of a highly selective group of patients who underwent AFCA. Second, the exact definition of the stiff LA syndrome included symptoms of the patients, such as dyspnea on exertion; however, since we could not obtain the data on symptoms, we designated stiff LA physiology. Furthermore, we estimated stiff LA physiology according to the RVSP change on echocardiography. However, several studies have defined LA stiffness using various parameters, such as LA peak pressure, pulmonary hypertension with large v-wave, and LA pulse pressure indicating LA compliance (Gibson et al., 2011; Witt et al., 2013; Park et al., 2015, 2019). Since there has been no gold-standard method for the LA stiffness, the associated results of each study could be different. In addition, applying the same criteria for stiff LA physiology in patients who already had diseased LA would be limited; therefore, the generalization of the results should be considered with circumspection. Third, patients with a lack of pre-procedural and appropriate follow-up echocardiography data were excluded. Thus, there could be a possibility of selection bias. In addition, since the post-ablation echocardiography was conducted 1 year after the procedure, other potential confounding factors, such as age, baseline comorbidities, prescribed medications, and inadequate rate control, could contribute to the progression of heart failure and eventually LA stiffness. Fourth, the echocardiography measurements could be inaccurate when measured during AF due to beat-to-beat variability. However, since all of the patients underwent serial echocardiography before and 1 year after the procedure, we could evaluate the changes in the parameters according to the development of stiff LA physiology. Fifth, in 6.7% of all the subjects, voltage mapping was performed after CPVI ablation, and it could have affected the mean LA voltage. Finally, although we waited for LA pressure to stabilize for at least 3 min in each patient, the mechanical stunning of LA after cardioversion may have affected the LA pressure.

## Conclusion

Although the incidence of post-AFCA stiff LA physiology was 3.7% and most of the cases were subclinical, the empirical extra-PV ablation was associated with this undesirable condition. In addition, patients who had low mean LA voltage before AFCA could be susceptible to stiff LA physiology.

## Data Availability Statement

The original contributions presented in the study are included in the article/supplementary material, further inquiries can be directed to the corresponding author/s.

## Ethics Statement

The studies involving human participants were reviewed and approved by The institutional Review Board of the Yonsei University Health System. The patients/participants provided their written informed consent to participate in this study.

## Author Contributions

J-HL and H-NP conceived and designed the study, performed the statistical analysis, and drafted manuscript. HY, T-HK, J-SU, BJ, M-HL, and H-NP recruited study subjects. O-SK developed customized software for calculating left atrial wall thickness and performed technical support. All authors have read and approved the final manuscript.

## Funding

This study was supported by the Ministry of Health and Welfare (Grant Nos. HI19C0114 and HI21C0011), and the National Research Foundation of Korea through the Basic Science Research Program (Grant No. NRF-2020R1A2B01001695), which is funded by the Ministry of Science, ICT, and Future Planning.

## Conflict of Interest

The authors declare that the research was conducted in the absence of any commercial or financial relationships that could be construed as a potential conflict of interest.

## Publisher's Note

All claims expressed in this article are solely those of the authors and do not necessarily represent those of their affiliated organizations, or those of the publisher, the editors and the reviewers. Any product that may be evaluated in this article, or claim that may be made by its manufacturer, is not guaranteed or endorsed by the publisher.
